# Endoscopic Ultrasound-Guided Treatments for Pancreatic Cancer: Understanding How Endoscopic Ultrasound Has Revolutionized Management of Pancreatic Cancer

**DOI:** 10.3390/cancers17010089

**Published:** 2024-12-30

**Authors:** Sahib Singh, Antonio Facciorusso, Rakesh Vinayek, Sudhir Dutta, Dushyant Singh Dahiya, Ganesh Aswath, Neil Sharma, Sumant Inamdar

**Affiliations:** 1 Department of Internal Medicine, Sinai Hospital of Baltimore, Baltimore, MD 21215, USA; 2 Gastroenterology Unit, Department of Medical and Surgical Sciences, University of Foggia, 71122 Foggia, Italy; 3 Clinical Effectiveness Research Group, Faculty of Medicine, Institute of Health and Society, University of Oslo, 0372 Oslo, Norway; 4 Division of Gastroenterology & Hepatology, Sinai Hospital of Baltimore, Baltimore, MD 21215, USA; 5 Division of Gastroenterology & Hepatology, Johns Hopkins Hospital, Baltimore, MD 21287, USA; 6 Division of Gastroenterology, Hepatology & Motility, The University of Kansas School of Medicine, Kansas City, KS 66160, USA; 7 Division of Gastroenterology & Hepatology, State University of New York Upstate Medical University, Syracuse, NY 13210, USA; 8 Division of Gastroenterology & Hepatology, Indiana University School of Medicine, Indianapolis, IN 46202, USA; 9 Department of Gastroenterology & Hepatology, University of Arkansas for Medical Sciences, Little Rock, AR 72205, USA; sumant.c.inamdar@gmail.com

**Keywords:** endoscopic ultrasound, biliary drainage, gallbladder drainage, gastroenterostomy, celiac plexus neurolysis, celiac ganglia neurolysis, radiofrequency ablation, fiducial markers, chemotherapy

## Abstract

Pancreatic cancer is associated with high rates of morbidity and mortality. Endoscopic ultrasound (EUS)-guided biopsy has become the standard diagnostic modality per the guidelines. The use of EUS has been growing for providing various treatments in patients with pancreatic cancers: biliary and gallbladder drainage for those with malignant biliary obstruction, gastroenterostomy for malignant gastric outlet obstruction, celiac plexus/ganglia neurolysis for pain control, radiofrequency ablation, placement of fiducial markers, and injection of local chemotherapeutic agents. In this review, we explore the recent clinical studies evaluating the EUS-guided treatments in pancreatic cancer.

## Simple Summary

Endoscopic ultrasound (EUS) involves study of the organs surrounding the gastrointestinal tract via use of ultrasound technology during upper endoscopy. In patients with suspected pancreatic cancer, EUS is advised to obtain biopsy for confirmation of diagnosis. With the recent advancements, EUS is now being utilized for certain treatments in pancreatic cancer patients, such as putting in stents to relieve blockage, injecting medicine to decrease pain, placing markers in pancreas for better radiation treatment, and destruction of the cancer cells with heat and local chemotherapy.

## 1. Introduction

Pancreatic cancer is one of the major causes of cancer related mortality in the United States, with only a 6% overall survival rate [1]. The risk factors include both genetic (such as Lynch syndrome) and non-genetic components (smoking and alcohol consumption). The evaluation of suspected pancreatic cancer involves imaging (computed tomography, magnetic resonance imaging, and positron emission tomography); diagnostic biopsy; and treatment (surgery, chemotherapy, radiotherapy, and symptomatic management). Of these, the only curative option is surgery; however, only 20% of patients have a resectable pancreatic cancer at diagnosis.

**Figure 1:**
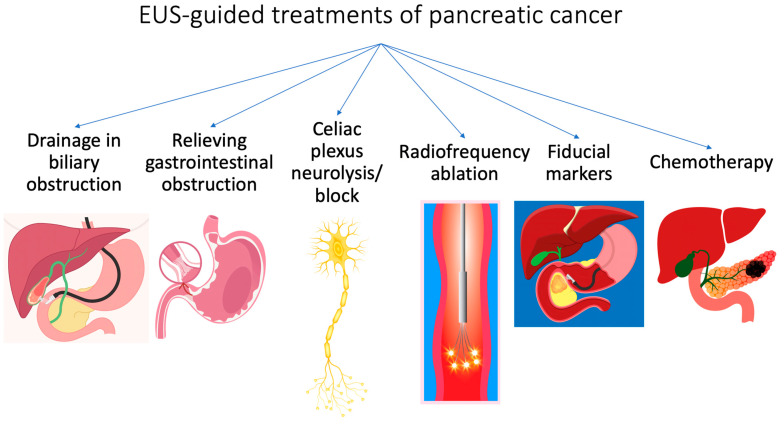
Major EUS-guided treatments in patients with pancreatic cancer.

Endoscopic modalities have had great advancements for the diagnosis and management of pancreatic cancers [2,3,4,5,6]. For locally advanced disease or borderline resectable disease on the initial imaging, endoscopic ultrasound (EUS)-guided biopsy is recommended by the European Society for Medical Oncology (ESMO) [7,8]. With regards to treatment, EUS-guided interventions have been widely studied for relieving obstruction (biliary or enteral using stents) and pain control (celiac plexus/ganglia neurolysis using local anesthetics and neurolytics) [9,10,11,12,13,14,15,16,17,18,19,20,21]. Other upcoming applications of EUS include radiofrequency ablation (RFA) of unresectable tumors, fiducial marker placement for accurate delivery of radiotherapy, and delivery of local chemotherapeutic agents [22]. In this review, we discuss the recent studies assessing the role of EUS in treatment of pancreatic cancer (Figure 1).

## 2. Biliary Obstruction

Among patients with pancreatic cancer-related malignant biliary obstruction, endoscopic retrograde cholangiopancreatography (ERCP) is the usual approach for biliary drainage. Guidelines have recommended EUS-guided biliary drainage (EUS-BD) over percutaneous transhepatic biliary drainage (PTBD) in cases with failed ERCP [23]. The primary methods for EUS-BD include EUS-assisted rendezvous (EUS-RV), EUS-guided hepaticogastrostomy (HGS), EUS-guided choledochoduodenostomy (CDS), and antegrade transpapillary or transanastomotic stent placement (EUS-AG) (Table 1) [24]. Given the rapid updates in endoscopic biliary drainage, the updated TOKYO criteria (2024) were released recently for assessing clinical outcomes, including terms such as ‘stent-demanding time’ [25].

In the prospective study by Krupa et al., liver function and quality of life (QoL) were assessed in patients undergoing EUS-BD for malignant biliary obstruction after failed ERCP [26]. Over the 14-day follow-up, EUS-BD led to improvement in serum liver function tests (*p* < 0.001), pruritus (*p* < 0.001), and anxiety and depression (*p* = 0.013) while also enabling chemotherapy resumption in 30% of patients. Vanella et al. conducted a multicenter study evaluating EUS-CDS with lumen apposing metal stents (LAMS) [27]. Of the 93 patients included, pancreatic cancer was present in 81% of cases. The clinical outcomes reported were technical success (97.8%), clinical success (93.4%), and adverse events (9.7%, with the majority [78%] being mild to moderate). LAMS dysfunction was observed in 31.8% of patients (mean follow-up 166 days), with the mean dysfunction-free survival of 394 days and the independent predictor of dysfunction being duodenal invasion. Endoscopic reintervention was able to correct the stent dysfunction in 96% of cases. As per-oral cholangioscopy, using a standard scope can be challenging in patients with distal malignant biliary strictures who have undergone EUS-CDS with LAMS, and suitable endoscopes such as a multibending ultra-slim endoscope are needed, as demonstrated by Yoo et al. [40]. EUS-guided interventions for drainage typically require fistula dilation before stent placement, which could lead to leakage of the contents in the abdominal cavity. Takeshita et al. evaluated EUS-guided intervention without fistula dilation using a novel laser-cut self-expandable metallic stent (SEMS) with a 7F delivery system in an attempt to reduce the adverse event rate [28]. Among the 11 patients enrolled, the overall success rate was 72.8%, of which EUS-BD had a 100% procedural success rate, with 57.1% in non-EUS-BD. Three patients (27.3%) had early adverse events: mild abdominal pain (2/11) and moderate bleeding (1/11). In the meta-analysis of 14 studies (620 participants) by Peng et al., EUS-BD with electrocautery-enhanced LAMS was assessed for palliation of malignant biliary obstruction after ERCP failure [29]. The analysis revealed a pooled technical success rate of 96.7%, clinical success of 91.0%, adverse events of 17.5%, and reintervention rate of 7.3%.

In a retrospective study by Kitagawa et al., EUS-HGS using dedicated plastic stents were used during the initial learning curve of this procedure [30]. In 23 patients where ERCP failed for biliary decompression, with most cases being of pancreatic cancer, EUS-HGS achieved a technical success rate of 95.7% and clinical success rate of 90.9%. Four patients (17.4%) reported adverse events: mild biliary peritonitis (3) and mild cholangitis (1), with no serious events occurring in any of the patients. Eight patients developed recurrent biliary obstruction, of which four underwent HGS stent replacement. Hedjoudje et al. evaluated the long-term effects of EUS-HGS for malignant biliary obstruction in 198 patients (pancreatic cancer in 49.5%), with the post-procedure median survival of 144 days (108–2011) [31]. Biliary obstruction was proximal in 68.4% of cases and distal in 27.6%. Around 33% patients had adverse events, with 19.1% developing recurrent biliary obstruction. Multivariate analysis revealed the use of partially covered self-expandable metal stents (PCSEMS) to be protective against recurrent obstruction (*p* = 0.034) and those with distal stenoses to have better stent patency (*p* = 0.031).

Sundaram et al. conducted a retrospective study of EUS-AG for preoperative/palliative biliary drainage in 54 patients (42.1% pancreatic cancer) who failed ERCP [32]. Around 64.8% of cases had a distal block and 35.1% had a proximal block. The technical success rate was 88.7% and clinical success rate was 95.7%. Patients stayed for a median duration of 1 day in the hospital after the procedure, with no procedure-related severe adverse events reported. Among the 20 patients who had EUS-AG for preoperative drainage, 95% had technical success and 94.5% had clinical success.

In order to improve upon the individual performances of EUS-HGS and EUS-AG, Inoue et al. conducted an observational study of 57 patients undergoing a combined procedure (EUS-HGAS) without fistula dilation for malignant distal biliary obstruction related to pancreatic cancer [33]. The procedural outcomes observed were technical success 91.2%, clinical success 91.2%, median procedural time 25 min, early adverse events 3.5%, and late adverse events 1.9%. Recurrent biliary obstruction was noted in 30.8% of cases, with the median time of development being 245 days and 100% of cases having successful endoscopic reintervention. Ishiwatari et al. compared EUS-HGS with EUS-HGAS in a propensity score matched study of 360 patients (81 matched pairs) with malignant distal biliary obstruction [34]. The two groups had comparable rates of adverse events (12.3% vs. 18.5%, *p* = 0.38) and overall survival (median 97 vs. 112 days, *p* = 0.88). However, the EUS-HGS group was associated with higher recurrent biliary obstruction (18 vs. 2 patients, *p* < 0.001), along with shorter development time (median 194 vs. 716 days, *p* < 0.01).

Several studies have now been published assessing EUS-BD as the primary modality for drainage compared to ERCP [41,42]. In the international multicenter study of 145 patients with malignant biliary obstruction scheduled for surgery, Tyberg et al. reported comparable rates of endoscopic clinical success (98% vs. 94%) and adverse events rates (17% vs. 26%) in the EUS-BD and ERCP groups [35]. The former had significantly higher surgical technical success (97% vs. 83%) and clinical success (97% vs. 75%), along with shorter hospital stay post-surgery (10 vs. 19 days, *p* = 0.0082). Chen et al. conducted a randomized trial of 144 patients with malignant distal biliary obstruction comparing EUS-CDS with LAMS vs. ERCP with metal stenting [36]. EUS-CDS had shorter procedure time (mean 14.0 [standard deviation 11.4] vs. 23.1 [15.6] minutes, *p* < 0.01), with comparable rates of technical success (90.4% vs. 83.1%), stent dysfunction (9.6% vs. 9.9%, *p* = 0.96), adverse events, oncologic outcomes, and quality of life. Barbosa et al. performed a meta-analysis of six randomized studies (577 patients) comparing EUS-BD and ERCP for drainage in malignant biliary obstruction [37]. The two groups were similar with regards to stent patency (mean difference [MD] 8.18 days), procedure time (MD −6.31 min), survival time (MD 4.59 days), technical success (93% vs. 88%), clinical success (89% vs. 88%), overall adverse events (8.33% vs. 15.61%), and cholangitis (2% vs. 2.55%). EUS-BD was better in terms of shorter hospital stay (MD −1.03 days), along with lower risk of reintervention (9.75% vs. 12.68%), post-procedure pancreatitis (0 vs. 5.83%), and tumor ingrowth/overgrowth (2.63% vs. 9.97%).

Palmieri conducted an online survey to identify the barriers to the adoption of EUS-BD for malignant distal biliary obstruction [43]. The majority of the respondents (115) were spread across North America (39.2%), Asia (28.6%), and Europe (20%). Only 10.5% respondents considered EUS-BD for first line drainage, owing to the lack of adequate data, risk of adverse events, and limited access to appropriate expertise. On the other hand, 40.9% of respondents favored EUS-BD over PTBD (21.7%) in cases of failed ERCP.

In patients where ERCP and EUS-BD are unsuccessful for drainage of malignant distal biliary obstruction, EUS-guided gallbladder drainage (EUS-GBD) has been evaluated [44]. In the multicenter retrospective study by Binda et al., 48 patients (pancreatic adenocarcinoma in 85.4%) underwent EUS-GBD with LAMS [38]. Over a mean follow-up of 122 days, the outcomes reported were technical success (100%), clinical success (81.3%), mean procedure time (26.4 min), mean hospital stay (9.2 ± 8.2 days), and adverse events (10.4%). Debourdeau et al. conducted a multicenter retrospective study comparing EUS-GBD and EUS-CDS after failed ERCP in 78 patients with malignant distal biliary obstruction (80.7% of cases due to pancreatic cancer) [39]. The two groups had similar rates of clinical success (87.8% vs. 89.2%, *p* = 0.8), technical success (100% vs. 94.6%, *p* = 0.132), periprocedural morbidity (<24 h) (9.8% vs. 13.5%, *p* = 0.368), time to recurrent biliary obstruction, and overall survival. The EUS-CDS group had a higher rate of late morbidity (>24 h) than EUS-GBD (21.6% vs. 7.3%, *p* = 0.042).

## 3. Gastrointestinal Obstruction

One of the other manifestations of pancreatic cancer is gastric outlet obstruction, for which surgical gastroenterostomy and endoscopic enteral stenting (ES) have been the usual treatment options. As EUS-guided gastroenterostomy (EUS-GE) showed favorable outcomes, it was included in the guidelines for the management of such patients in expert settings [23,45,46]. In the meta-analysis by Kumar et al., EUS-GE showed lower odds of technical success but higher clinical success, lower adverse events rate, and shorter procedure time and post-procedure length of stay when compared to surgical gastroenterostomy [47].

De Ponthaud et al. conducted an online survey of 290 pancreatologists (35% surgeons, 65% gastroenterologists) regarding EUS-Gastrojejunostomy (EUS-GJ) in patients with malignant gastric outlet obstruction due to pancreatic cancer [48]. ES was the most commonly reported treatment option (86%), followed by surgical GJ (76%). Even though EUS-GJ was available to 59% of physicians, only 10% had expertise in this technique. Around 51% respondents agreed that EUS-GJ will likely be the primary treatment for outlet obstruction in the future. On the other hand, 19% reported that EUS-GJ would be used only after ES failure. The respondents concluded that, while EUS-GJ is minimally invasive with good clinical efficacy, the adoption is limited due to the steep learning curve.

In the prospective matched cohort study by Vanella et al., patients with malignant gastric outlet obstruction were treated with either EUS-GE or ES (28 patients each), with the former having a higher clinical success rate (100% vs. 75.0%, *p* = 0.006), lower recurrence rate (3.7% vs. 33.3%, *p* = 0.02), and possible shorter time to chemotherapy [49]. Conti Bellocchi et al. conducted a retrospective propensity score-matched study comparing EUS-GE vs. ES in 198 patients with malignant gastric outlet obstruction [50]. EUS-GE had a lower rate of stent dysfunction (3.1% vs. 16.9%, *p* = 0.004). After matching (45 patients in each group), both groups showed a 100% technical success rate. While stent dysfunction was higher in the ES group (20% vs. 4.4%, *p* = 0.022), there was no difference in clinical efficacy (*p* = 0.266) or safety (*p* = 0.085). EUS-GE had a shorter hospital stay (7.5 vs. 12.5 days, *p* = 0.018).

## 4. Celiac Plexus Neurolysis/Block

Pancreatic cancer can often be associated with abdominal pain, necessitating the use of nonsteroidal anti-inflammatory drugs and opioid analgesics, leading to increased risk of side effects related to these medications [51]. EUS-guided interventions such as the injection of local anesthetics (celiac block), neurolytic agents like ethanol (celiac plexus neurolysis [EUS-CPN]) and RFA of the celiac ganglia have shown great results in improving pain control and quality of life [52,53,54].

Han et al. conducted a retrospective study of 58 patients with pancreatic cancer undergoing EUS-CPN and reported a good pain response in 74.1% of patients at 1 week and 67.2% of patients at 4 weeks [55]. Patients with invisible ganglia (at 1 week), metastatic disease (4 weeks), and invasion of the celiac plexus were found to be significant factors for a negative response via the multivariate analysis. In the multicenter prospective trial by Kamata et al., EUS-CPN and EUS-celiac ganglia neurolysis (CGN) showed technical success rates of 100% and 80.4%, respectively, with the overall efficacy rate of 82.4% and complete pain relief rate of 27.4% [56]. Around 15.7% of patients had adverse events. EUS-CPN plus EUS-CGN had a higher efficacy rate and complete pain relief than EUS-CPN alone. In a retrospective study by Liu et al., nursing cooperation patterns were evaluated for patients with advanced pancreatic cancer who underwent EUS-CPN [57]. Implementation of a quality control research group during EUS-CPN led to higher nurses’ satisfaction (*p* < 0.001), with a possible reduction in procedure times.

The type of local anesthetics used can also have an impact on clinical outcomes. In the retrospective multicenter study by Zhao et al., bupivacaine and ropivacaine were compared for EUS-CPN [58]. The 0.75% bupivacaine group had the lowest incidence of procedure-related pain within 12 h after EUS-CPN (10.38%, *p* = 0.04), as compared to the ropivacaine group (26.09%) and 0.375% bupivacaine group (23.81%). On the other hand, post-procedural arrhythmia was lower in the ropivacaine group (13.04%) than the 0.375% bupivacaine and 0.75% bupivacaine groups (19.05% and 18.87%). CNS toxicity was not reported in any of the patients. Saleh et al. assessed the addition of dexmedetomidine to 0.5% bupivacaine for EUS-CPN in patients with pancreatic cancer [59]. The combination resulted in a greater degree and duration of pain relief as compared to bupivacaine alone, without any difference in overall survival.

In the network meta-analysis of 10 RCTs (662 pancreatic cancer patients) by Okita et al., EUS-CPN + medical management was found to be better in improving pain control as compared to medical management alone (MD −1.30) and the percutaneous CPN (P-CPN) + medical management groups (MD −0.88) [60]. Koulouris et al. conducted a meta-analysis of 17 studies evaluating EUS-CPN in pancreatic cancer [61]. The overall response rate was 68% and 53% at 2 and 4 weeks, respectively, without any significant difference between central injection, bilateral injection, and CGN techniques. Serious complications were reported in the bilateral injection and CGN groups. In another meta-analysis by Asif et al., 16 studies evaluating EUS-CPN for pancreatic cancer-related pain were analyzed [62]. Around 71% of patients reported pain relief with EUS-CPN: 66% with the central and 57% with the bilateral technique. Li et al. conducted a qualitative systematic review comparing the EUS-CGN and EUS-CPN techniques [63]. Among the five studies included with 319 patients, EUS-CGN had 65.0% to 88.46% short-term pain response rates, which were higher than EUS-CPN. Adverse events such as transient hypotension and gastrointestinal symptoms were comparable.

## 5. Radiofrequency Ablation

EUS-RFA has become a popular therapeutic modality in clinical studies for pancreatic cystic lesions, neuroendocrine tumors, and, more recently, pancreatic cancers in patients not suitable for undergoing surgery [64,65]. RFA is performed via a FNA needle using special electrodes to convert radiofrequency waves into heat (350–500 kHz), thereby inducing thermal coagulation and tissue destruction [66,67,68,69]. Clinical studies have shown improved survival in patients treated with EUS-RFA when used with palliative surgeries [70,71,72,73,74,75]. RFA not only treats tumors directly but also stimulates the immune system by changing the tumor environment and increasing immune cell activity [76].

Robles-Medranda et al. conducted an observational study of EUS-RFA for patients with unresectable pancreatic ductal adenocarcinoma [77]. Among the 26 patients in the study (15 with locally advanced and 11 with metastatic neoplasm), technical success was reported in all without any major adverse events. The overall survival rate was 42.3% at 6 months, with significant improvement in the performance status (*p* = 0.03). Metastatic disease was associated with worse overall survival (*p* = 0.004).

Kongkam et al. evaluated EUS-RFA plus systemic chemotherapy (10 patients) vs. chemotherapy alone (12 patients) for pancreatic cancer [78]. Technical success was achieved with all the EUS-RFA procedures. Although no significant difference was observed between the groups with respect to the pre-treatment and post-treatment mean maximal tumor diameters, the chemotherapy alone group had a significant increase in size (50.5 mm to 56.3 mm, *p* = 0.017). The combined treatment group had a higher rate of tumor necrosis (100% vs. 50%, *p* = 0.014), with significant reduction in the mean narcotic pain drug dosage (63.6 mg to 37.1 mg, *p* = 0.022). The mortality rates at 6 months were comparable in the two groups.

## 6. Fiducial Markers

The successful placement of fiducial markers via EUS is another upcoming application for the accurate delivery of radiotherapy in patients with pancreatic tumors [79,80,81]. In a retrospective study of 82 patients by Cazacu et al., 230 fiducial markers were placed under guidance by EUS, with a technical success rate of 98% and no immediate adverse events [82]. After stereotactic body radiotherapy (SBRT), 41% reported no toxicities, while 35% reported fatigue and nausea. In another prospective study by Figueiredo et al., 37 pancreatic cancer patients underwent 97 EUS-guided fiducial placements [83]. Technical success was reported in 92% of cases, with high-quality success in 62.5% and 8% having adverse events such as mild acute pancreatitis, fever, and biliary stent migration. In the prospective trial by Chang et al., EUS-guided fiducial marker placement was performed to guide pancreatic surgery in 20 patients [84]. As compared to the patient group without a marker, the EUS group showed improved detection during the surgery (*p* = 0.011).

## 7. Chemotherapy

EUS-guided delivery of chemotherapeutic agents provides a minimally invasive therapeutic option [85,86,87]. Sharma et al. conducted a study of EUS—fine needle injection of large surface area microparticle paclitaxel in patients with unresectable locally advanced pancreatic cancer [88]. After the intra-tumoral chemotherapy injection, the tumors became resectable in eight patients. Immunofluorescence of the resected specimens showed increased T cells, natural killer cells, and macrophages, with decreased myeloid-derived suppressor cells. Around 94% of patients demonstrated disease control at 6 months, with the overall survival being 19.7 months for patients who received two injections.

## 8. Miscellaneous Applications

Yamashita et al. utilized contrast-enhanced harmonic EUS (CH-EUS) for prediction of the treatment efficacy of neoadjuvant chemotherapy via study of the pathological response [89]. The CH-EUS enhancement pattern could be used as an indication for neoadjuvant chemotherapy, while the CH-EUS vascular pattern can predict its efficacy. Ashida et al. demonstrated EUS-guided high-intensity focused ultrasound (EUS-HIFU) ablation in a preclinical model [90]. Bhutani et al. evaluated the feasibility of EUS-guided polyethylene glycol hydrogel injection between the pancreatic head and duodenum to allow for enhanced radiotherapy [91]. Similar to RFA, EUS-guided cryoablation, ethanol ablation, and thermal ablation were also reported in clinical studies recently [92,93,94].

## 9. Limitations and Future Perspectives

Although EUS procedures provide several advantages, there are some limitations to their use as well. EUS based interventions are technically difficult, requiring skilled endoscopists with the expertise to perform them. There is limited availability of the specialized devices for EUS as compared to conventional ERCP, which is widely available. Additionally, the associated costs for EUS may be higher than other procedures. Finally, there is a lack of adequate data for some of the newer approaches. Future studies, especially randomized trials, would be needed to establish the role of EUS in pancreatic cancer.

## 10. Conclusions

Aside from diagnostic utility, EUS-guided interventions have undergone rapid advancements for various treatments in patients with pancreatic cancers, such as for relieving biliary/enteral obstruction using stent placement, celiac plexus/ganglia neurolysis to achieve pain control, RFA of unresectable tumors, fiducial marker placement, and application of local chemotherapy. EUS provides a promising future with great efficacy and low adverse event rates in this patient population.

## Figures and Tables

**Table 1:** Clinical studies assessing EUS-guided drainage in patients with biliary obstruction.

Study	Type	Number of Patients	Clinical Outcomes
Krupa et al. [26]	Observational	37	EUS-BD: improvement in serum liver function tests (*p* < 0.001), pruritus (*p* < 0.001), anxiety and depression (*p* = 0.013)
Vanella et al. [27]	Observational	93	EUS-CDS with LAMS: technical success (97.8%), clinical success (93.4%), adverse events (9.7%, with the majority [78%] being mild to moderate)
Takeshita et al. [28]	Observational	11	EUS-guided intervention without fistula dilation using a novel laser-cut SEMS: overall success rate 72.8% (EUS-BD had a 100% procedural success rate, with 57.1% in non-EUS-BD), early adverse events (27.3%)
Peng et al. [29]	Meta-analysis	620	EUS-BD with electrocautery-enhanced LAMS: technical success (96.7%), clinical success (91.0%), adverse events (17.5%), reintervention rate (7.3%)
Kitagawa et al. [30]	Observational	23	EUS-HGS using dedicated plastic stents: technical success (95.7%), clinical success (90.9%), adverse events (17.4%)
Hedjoudje et al. [31]	Observational	198	EUS-HGS: adverse events (33%), recurrent biliary obstruction (19.1%)
Sundaram et al. [32]	Observational	54	EUS-AG: technical success (88.7%), clinical success (95.7%), no procedure-related severe adverse events
Inoue et al. [33]	Observational	57	EUS-HGAS without fistula dilation: technical success (91.2%), clinical success (91.2%), median procedural time (25 min), early adverse events (3.5%), late adverse events (1.9%), recurrent biliary obstruction (30.8%)
Ishiwatari et al. [34]	Observational	360	EUS-HGS vs. EUS-HGAS: adverse events (12.3% vs. 18.5%, *p* = 0.38), overall survival (median 97 vs. 112 days, *p* = 0.88), recurrent biliary obstruction (18 vs. 2 patients, *p* < 0.001)
Tyberg et al. [35]	Observational	145	EUS-BD vs. ERCP: endoscopic clinical success (98% vs. 94%), adverse events rates (17% vs. 26%)
Chen et al. [36]	Randomized trial	144	EUS-CDS with LAMS vs. ERCP with metal stenting: procedure time (14.0 [11.4] vs. 23.1 [15.6] minutes, *p* < 0.01), technical success (90.4% vs. 83.1%), stent dysfunction (9.6% vs. 9.9%, *p* = 0.96)
Barbosa et al. [37]	Meta-analysis	577	EUS-BD vs. ERCP: stent patency (MD 8.18 days), procedure time (MD −6.31 min), survival time (MD 4.59 days), technical success (93% vs. 88%), clinical success (89% vs. 88%), overall adverse events (8.33% vs. 15.61%), cholangitis (2% vs. 2.55%), hospital stay (MD −1.03 days), reintervention (9.75% vs. 12.68%), post procedure pancreatitis (0 vs. 5.83%), tumor ingrowth/overgrowth (2.63% vs. 9.97%)
Binda et al. [38]	Observational	48	EUS-GBD with LAMS: technical success (100%), clinical success (81.3%), mean procedure time (26.4 min), mean hospital stay (9.2 ± 8.2 days), adverse events (10.4%)
Debourdeau et al. [39]	Observational	78	EUS-GBD vs. EUS-CDS: clinical success (87.8% vs. 89.2%, *p* = 0.8), technical success (100% vs. 94.6%, *p* = 0.132), periprocedural morbidity (<24 h) (9.8% vs. 13.5%, *p* = 0.368)

EUS-BD—endoscopic ultrasound (EUS)-guided biliary drainage, EUS-CDS—EUS-guided choledochoduodenostomy, LAMS—lumen apposing metal stents, EUS-HGS—EUS-guided hepaticogastrostomy, EUS-AG—EUS-guided antegrade stent placement, EUS-HGAS—EUS-guided hepaticogastrostomy with antegrade stenting, ERCP—endoscopic retrograde cholangiopancreatography, MD—mean difference, and EUS-GBD—EUS-guided gallbladder drainage.

## Data Availability

The data presented in the study are openly available in online databases.
